# Association between triglyceride-glucose-related indices and cardiovascular disease risk in population with cardio-kidney-metabolic syndrome stages 0–3: a systematic review and meta-analysis

**DOI:** 10.1186/s12872-026-06534-w

**Published:** 2026-08-26

**Authors:** Yanxu Chen, Bo Dai, Jieyu Zhang, Zhenru Liu, Haotian Qi, Yang Xiao, Hezeng Dong, Ziqiang Chen, Qinghai Long, Shengxian Xu, Jian Sun

**Affiliations:** 1 College of Science and Technology Changchun, Changchun, China; 2 Changchun University of Chinese Medicine, Changchun, China

**Keywords:** Cardiovascular diseases, Cardiovascular risk, Triglyceride-glucose index, Insulin resistance, Cohort studies, CKM syndrome

## Abstract

**Objective:**

This systematic review and meta-analysis aimed to compare the associations of four triglyceride-glucose (TyG)-derived indices (TyG, TyG-BMI, TyG-WC, TyG-WHtR) with incident cardiovascular disease (CVD) risk among individuals with stage 0–3 cardio-kidney-metabolic (CKM) syndrome, and explore potential sources of inter-study heterogeneity using pre-specified subgroup analyses and exploratory meta-regression analyses.

**Methods:**

We systematically searched PubMed, Embase, Web of Science, and the Cochrane Library from database inception to May 1, 2026. Two independent reviewers completed study screening, data extraction, and quality assessment using the Newcastle–Ottawa Scale (NOS). Pooled hazard ratios (HRs) with 95% confidence intervals (CIs) were synthesized using fixed- or random-effects models according to between-study heterogeneity, which was quantified by Cochran’s Q test and the I² statistic. Subgroup analyses, exploratory univariable meta-regression analyses, leave-one-out sensitivity analyses, and publication bias assessments were performed to investigate potential sources of heterogeneity and evaluate the robustness of the findings.

**Results:**

Nine high-quality prospective cohort studies were ultimately included. Participants in the highest versus lowest quantiles of each index had significantly elevated CVD risk: TyG (HR = 1.24, 95% CI 1.16–1.33, I² = 76%), TyG-BMI (HR = 1.44, 95% CI 1.26–1.66, I² = 93%), TyG-WC (HR = 1.42, 95% CI 1.21–1.67, I² = 96%), and TyG-WHtR (HR = 1.45, 95% CI 1.28–1.65, I² = 91%). Exploratory meta-regression suggested that follow-up duration may contribute to heterogeneity in TyG, whereas age may contribute to heterogeneity in TyG-WHtR; substantial residual heterogeneity remained for TyG-WC and TyG-WHtR. Sensitivity analyses supported the robustness of the findings. No significant publication bias was detected, but statistical power was limited by fewer than 10 studies per index.

**Conclusions:**

This meta-analysis suggests that elevated TyG-derived indices are significantly associated with increased incident CVD risk among individuals with stage 0–3 CKM syndrome. Composite TyG-derived indices showed numerically higher pooled effect estimates than the TyG index; however, overlapping confidence intervals and the absence of direct statistical comparisons prevent conclusions regarding the superiority of any specific index. Given the observational nature of the included studies, limited population diversity, and substantial residual heterogeneity, these findings should be interpreted cautiously. TyG-derived indices may represent potential auxiliary markers for CVD risk stratification, and further large-scale multi-ethnic prospective studies are warranted to evaluate their clinical utility.

**Graphical Abstract:**

**Figure:**
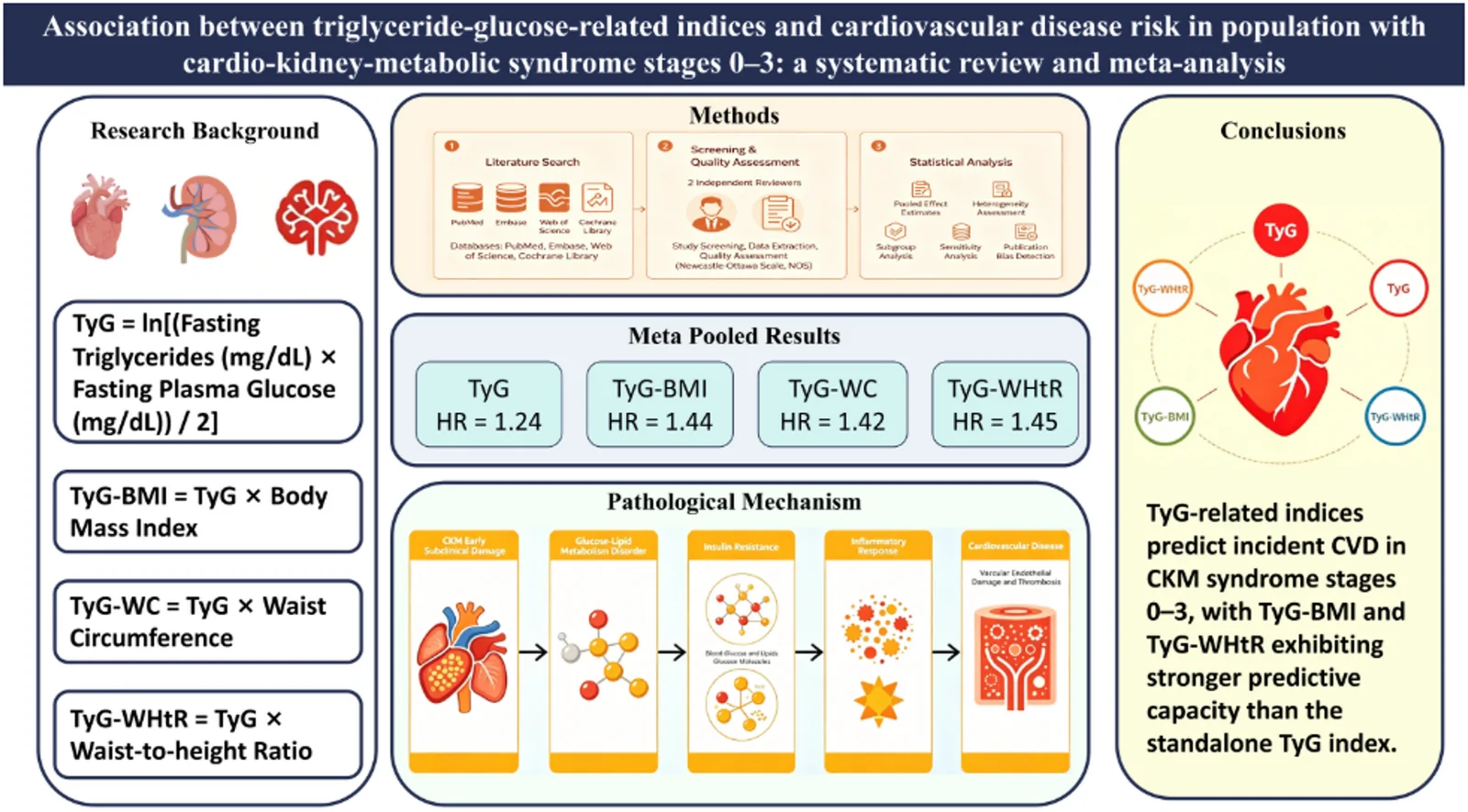

**Supplementary Information:**

The online version contains supplementary material available at https://doi.org/10.1186/s12872-026-06534-w.

## Introduction

Cardio-kidney-metabolic (CKM) syndrome is a systemic disorder characterized by the interconnected pathophysiology of metabolic dysfunction, chronic kidney disease, and cardiovascular disease (CVD) [1, 2]. Stages 0–3 represent the early stages of CKM syndrome and are associated with an increased risk of future cardiovascular events [3]. Despite the absence of overt clinical manifestations, individuals with early-stage CKM may already have increased CVD risk, posing a growing global public health burden [4]. Conventional CVD risk prediction scores are mainly based on established clinical risk factors and may not fully capture subclinical metabolic and renal abnormalities, highlighting an unmet need for low-cost, readily available, and reliable biomarkers to facilitate early cardiovascular risk stratification in this high-risk population [5].

Insulin resistance (IR) is considered an important pathophysiological mechanism linking CKM syndrome with adverse cardiovascular outcomes [6]. The triglyceride-glucose (TyG) index and its anthropometric-derived indicators, including TyG-body mass index (TyG-BMI), TyG-waist circumference (TyG-WC), and TyG-waist-to-height ratio (TyG-WHtR), have been widely used as convenient noninvasive surrogate markers of systemic IR [7, 8]. Several prospective cohort studies have investigated the associations of these TyG-related indices with incident CVD specifically among individuals with stage 0–3 CKM syndrome. Nevertheless, the reported effect estimates remain inconsistent across independent investigations, with notable discrepancies among studies [9]. Between-study heterogeneity arising from variations in participant characteristics, follow-up duration, study design, and confounding adjustment strategies has not been comprehensively evaluated, limiting a clear understanding of the potential clinical utility of these metabolic biomarkers [10].

Accordingly, this systematic review and meta-analysis synthesizes evidence from available prospective cohort studies to quantitatively evaluate the associations of four TyG-derived indices (TyG, TyG-BMI, TyG-WC, and TyG-WHtR) with incident CVD risk among individuals with stage 0–3 CKM syndrome. Pre-specified subgroup analyses were performed to explore potential drivers of between-study heterogeneity, while leave-one-out sensitivity analyses and publication bias assessments were conducted to evaluate the robustness of pooled effect estimates. This study aims to systematically evaluate the associations of these indices with incident CVD risk and explore their potential utility as auxiliary markers for preliminary CVD risk stratification.

## Methods

The present systematic review and meta-analysis was prospectively preregistered on the International Prospective Register of Systematic Reviews (PROSPERO; Registration No. CRD420261406283) and conducted in accordance with the 2020 Preferred Reporting Items for Systematic Reviews and Meta-Analyses (PRISMA) reporting guidelines [11]. The completed PRISMA checklist is provided in Supplementary Material S1.

### Data sources and search strategy

A systematic literature search was conducted across four electronic databases: PubMed, Embase, Web of Science, and the Cochrane Library, from database inception to May 1, 2026. Key search terms covered three domains: (1) target population: cardio-kidney-metabolic syndrome, CKM syndrome, CKM; (2) exposure markers: triglyceride-glucose index, TyG index, TyG-related indices, TyG-BMI, TyG-WC, and TyG-WHtR; (3) outcomes: cardiovascular disease, CVD, coronary heart disease, stroke, myocardial infarction, and major adverse cardiovascular events (MACE); and (4) study design: cohort study and prospective cohort study. Cross-sectional studies were retained in the initial search strategy to ensure retrieval comprehensiveness but were excluded during full-text screening because they generally cannot provide appropriate data on incident CVD outcomes. The detailed search strategy is provided in Supplementary Material S2.

### Study selection criteria

Eligibility criteria were formulated in strict accordance with the PECOS (Population, Exposure, Comparator, Outcome, Study design) framework.

#### Participants

Studies enrolling individuals classified as having stage 0–3 CKM syndrome according to the criteria used in each cohort were eligible.

#### Exposure

Studies reporting at least one of the four TyG-derived indices were included. The standardized calculation formulas for each index are as follows:

- TyG = ln[(fasting triglycerides (mg/dL) × fasting plasma glucose (mg/dL))/2]
- TyG-BMI = TyG × body mass index
- TyG-WC = TyG × waist circumference
- TyG-WHtR = TyG × waist-to-height ratio

#### Comparators

Studies comparing the highest versus lowest quantiles (e.g., quartiles, tertiles) of TyG-related indices, with the lowest quantile clearly defined as the reference group, were eligible.

#### Outcomes

The primary outcome was incident (new-onset) CVD, including coronary heart disease, stroke, and myocardial infarction. All included studies were required to provide clearly defined criteria for identifying CVD events. Studies providing extractable hazard ratios (HRs) with corresponding 95% confidence intervals (CIs) for the association between TyG-related indices and incident CVD risk were included; multivariable-adjusted HR estimates were prioritized over crude unadjusted values.

#### Study design

Only peer-reviewed, full-text prospective cohort studies published in English were eligible for final quantitative synthesis. Cross-sectional studies were retained in the initial search strategy to ensure retrieval comprehensiveness but were excluded during full-text screening because they generally cannot provide appropriate data on incident CVD outcomes.

### Exclusion criteria

#### Types of participants

Studies were excluded if participants had pre-existing CVD at baseline, end-stage renal disease, dialysis dependence, renal transplantation, severe chronic liver disease, malignant tumors, or other severe comorbidities that could substantially influence metabolic status or cardiovascular outcomes.

#### Exposure

Studies without available data on TyG, TyG-BMI, TyG-WC, or TyG-WHtR, or those reporting only non-TyG metabolic markers, were excluded.

#### Comparators

Studies without clearly defined comparison groups based on quantiles of TyG-related indices, or those using arbitrary cut-off values rather than quantile-based categories, were excluded.

#### Outcome measures

Studies focusing on prevalent rather than incident CVD, using unclear definitions of CVD events, or lacking extractable HRs with corresponding 95% CIs were excluded.

#### Type of study

Case reports, narrative reviews, previous meta-analyses, letters, editorials, conference abstracts, randomized controlled trials, cross-sectional studies, retrospective cohort studies, animal experiments, and in vitro studies were excluded.

### Literature screening and data extraction

Two independent reviewers (Yanxu Chen, Hezeng Dong) performed literature screening and data extraction independently and in duplicate, strictly following the pre-specified eligibility criteria. Duplicate records were first removed using EndNote X9. Titles and abstracts of the remaining records were then independently screened to exclude obviously irrelevant studies. Subsequently, full texts of potentially eligible citations were retrieved and assessed in detail to determine final inclusion. Any unresolved discrepancies during screening and extraction were resolved via group discussion or arbitration by a third senior investigator (Ziqiang Chen).

A standardized data extraction form was used to collect key study information, including first author, publication year, study location, study design, total sample size, mean/median age, male proportion of participants, diagnostic criteria and operational definitions of stage 0–3 CKM syndrome, baseline levels of TyG-derived indices, follow-up duration, definition of incident CVD, adjusted confounding variables, and crude or multivariable-adjusted HRs with corresponding 95% CIs. The CKM staging criteria applied in each included study were carefully reviewed and recorded to assess potential variations in the operationalization of CKM syndrome across cohorts. All extracted data were independently cross-validated by two additional researchers (Bo Dai, Qinghai Long) to ensure data accuracy and completeness.

### Quality assessment

The Newcastle–Ottawa Scale (NOS) for cohort studies was used to evaluate the methodological quality of all included prospective cohort studies, with a total possible score ranging from 0 to 9 [12]. The scale comprises three core domains: selection of study cohorts, comparability of cohorts, and ascertainment of outcomes. Studies with a total score of ≥ 7 were classified as high-quality, whereas those with a score < 7 were considered low-quality. Quality assessment was independently conducted in duplicate by two researchers (Yanxu Chen, Hezeng Dong). Any discrepancies in scoring were resolved through consensus discussion or arbitration by a third senior investigator (Ziqiang Chen).

### Statistical analysis

All statistical analyses were performed using Review Manager 5.4.1 and Stata 16.0 software. Multivariable-adjusted HRs with 95% CIs were used as the pooled effect estimates to quantify the association between TyG-derived indices and incident CVD risk. Pooled estimates were synthesized by comparing participants in the highest versus lowest quantiles of each index across included studies.

Between-study heterogeneity was assessed using Cochran’s Q test (*P* < 0.10 considered statistically significant) and quantified using the I² statistic. According to the Cochrane Handbook for Systematic Reviews of Interventions (7th edition), I² values of 0%–40%, 30%–60%, 50%–90%, and 75%–100% were considered as potentially unimportant, moderate, substantial, and considerable heterogeneity, respectively [13]. A fixed-effects model was applied when heterogeneity was low (I² ≤ 40%), whereas a random-effects model was used when substantial heterogeneity was observed (I² > 50% or *P* < 0.10).

Pre-specified subgroup analyses were conducted according to participant age, follow-up duration, and total sample size to explore potential sources of heterogeneity. Pooled HRs were estimated separately within each subgroup, and differences between subgroups were evaluated using the Q test for subgroup differences.

Leave-one-out sensitivity analyses were performed to evaluate the robustness of the pooled estimates by sequentially removing individual studies. Publication bias was assessed using funnel plots and Egger’s linear regression test when applicable, with *P* < 0.05 considered statistically significant [14]. However, the interpretation of publication bias tests was cautious because fewer than 10 studies were included for each TyG-derived index.

Given the substantial heterogeneity observed across studies, exploratory univariable meta-regression analyses were further performed using a restricted maximum likelihood (REML) approach to investigate potential study-level moderators of heterogeneity. Participant age, follow-up duration, and total sample size were included as potential moderators. Due to the limited number of studies available for each index, multivariable meta-regression models including multiple moderators were not performed because of the potential risk of overfitting and unstable regression estimates.

## Results

### Literature screening results

**Fig. 1:**
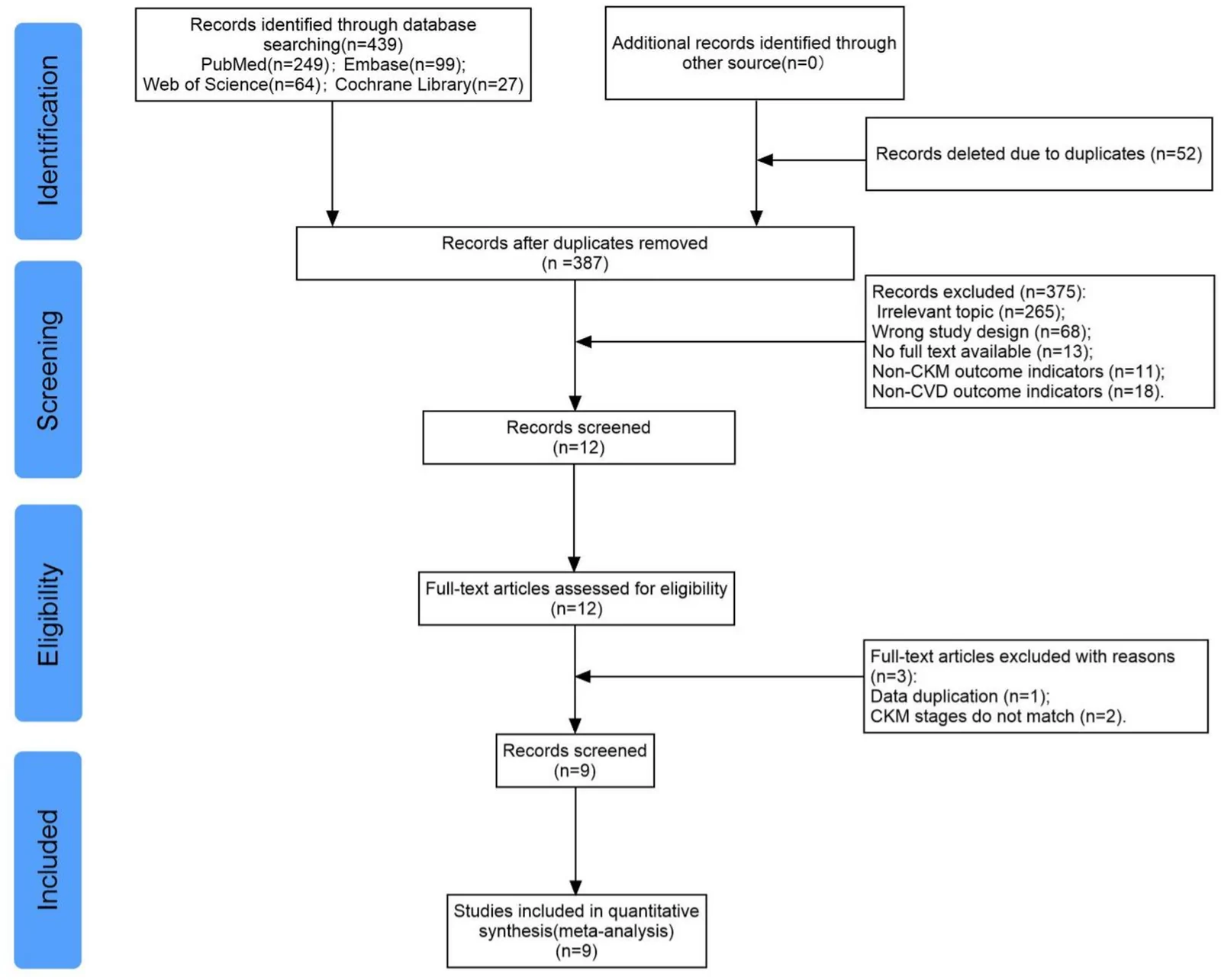
Flowchart of study selection

A total of 439 records were initially retrieved from four electronic databases (PubMed, Embase, Web of Science, and the Cochrane Library), with no additional records identified through other sources. After removing 52 duplicate records, 387 unique citations remained for title and abstract screening. During this stage, 375 records were excluded for reasons including irrelevant topics, ineligible study designs, unavailable full-text articles, and non-target exposures, leaving 12 studies for full-text eligibility assessment. Three studies were further excluded after full-text review: two due to overlapping study populations and one that did not meet the predefined CKM classification criteria. Ultimately, nine prospective cohort studies met all pre-specified eligibility criteria and were included in the final meta-analysis [15–23]. The screening flowchart is presented in Fig. 1.

### Characteristics of included studies

All nine included studies were prospective cohort studies published between 2024 and 2026. Eight studies were conducted in China and one in the United Kingdom. The sample size of individual studies ranged from 3,948 to 463,545, with a total combined sample of 789,525 individuals classified as having stage 0–3 CKM syndrome across all cohorts. The mean or median age of participants ranged from 57.0 to 70.82 years, and the proportion of male participants varied from 39.60% to 52.62%. The follow-up duration spanned 5.0 to 13.6 years across all included cohorts. All studies evaluated the associations of TyG-derived indices with incident CVD risk and provided multivariable-adjusted HRs with corresponding 95% CIs based on comparisons of the highest versus lowest quantiles of each biomarker. The adjusted confounding variables varied across studies, mainly covering demographic characteristics, lifestyle factors, chronic metabolic diseases, and conventional laboratory parameters, which represented potential sources of between-study heterogeneity. Detailed baseline characteristics of all enrolled studies are summarized in Table 1.

**Table 1:** Basic characteristics of the included studies

Number	Study	Country	Age (year)	Male (%)	Numberofparticipants	TyGrelatedindices	Follow-uptime	HR (95%CI)	Studydesign	Typeofoutcome	Variablesadjusted
1	Weipeng Li 2024 [15]	China	Average age: 59.17 ± 9.28 years	52.62%	7,376	TyG-BMI	8 years	1.798(1.410–2.294)	Prospective cohort study	CVD	Age, gender, Smoking status, Drinking status, Sleep problems, Education level, Marital status, BUN, Scr, TC, HDL-C, LDL-C, CRP, UA, PLT, Hypertension, Diabetes, Depression.
2	Kun Liu 2025 [16]	UK	Median age: 57.0 years	46.80%	282,920	TyG; TyG-BMI; TyG-WC; TyG-WHtR.	13.6 years	1.30 (1.25–1.35); 1.49 (1.43–1.55); 1.58 (1.51–1.65); 1.58 (1.51–1.65).	Prospective cohort study	CVD	Age, gender, ethnicity, employed status, Townsend deprivation index, educational level, family income, smoking status, alcohol drinking, sleep duration, physical activity, diet quality, systolic/diastolic blood pressure, glycated hemoglobin, total cholesterol, and low-density lipoprotein cholesterol.
3	Jianan Hong 2025 [17]	China	Average age: 60.53 ± 9.33 years	46.90%	7,364	TyG; TyG-BMI; TyG-WC; TyG-WHtR.	9 years	1.21 (1.15–1.28); 1.23 (1.16–1.29); 1.20 (1.14–1.27); 1.65 (1.40–1.94).	Prospective cohort study	CVD	Age, gender, education levels, marital status, smoking and drinking status and history of hypertension, diabetes, BUN, UA, eGFR and LDL.
4	Yilin Pan 2025 [18]	China	Median age: 60.0 years	46.30%	5,782	TyG; TyG-BMI; TyG-WC; TyG-WHtR.	6 years	1.13 (0.95–1.35); 1.50 (1.25–1.80); 1.42 (1.19–1.70);1.36 (1.14–1.64).	Prospective cohort study	CVD	Age, gender, marital status, education, residence, smoking, drinking, hypertension, diabetes mellitus, dyslipidemia, creatinine, and CRP.
5	Menghe Wang 2025 [19]	China	Average age: 70.82 ± 6.03 years	39.60%	463,545	TyG; TyG-BMI; TyG-WC; TyG-WHtR.	5 years	1.150 (1.091–1.212); 1.116 (1.037–1.202) ;1.190 (1.121–1.264) ;1.177 (1.106–1.251) .	Prospective cohort study	CVD	Age, gender, education, marriage status, occupation, smoking, drinking, physical activity, and BMI.
6	Jintao Chen 2025 [20]	China	Average age: 61.78 ± 8.68 years	45.7%	5,111	TyG	10 years	1.36(1.03–1.79)	Prospective cohort study	CVD	Age, gender, obesity, education level, marital status, residence, smoking, drinking, antihypertensive drugs, antidiabetic drugs, TC, LDL-C, lung diseases, liver diseases, cancer, and digestive disease.
7	Cheng Huang 2025 [21]	China	Average age: 58.47 ± 9.50 years	46.48%	6,764	TyG-WHtR	7.77 ± 2.13 years	1.28 (1.07–1.54)	Prospective cohort study	CVD	Age, gender, marital status, rural, hypertension, dyslipidemia, diabetes, drinking status, smoking status, CKM, medication history, SBP, DBP, HDL-C, LDL-C, TC, UA, BUN, HbA1c and CRP.
8	Shu-Shu Han 2026 [22]	China	Average age: 58.80 ± 8.8 years	47.0%	3,948	TyG; TyG-BMI; TyG-WC; TyG-WHtR.	8 years	1.57 (1.27–1.93); 1.95 (1.57–2.42); 1.97 (1.59–2.44); 1.83 (1.47–2.27).	Prospective cohort study	CVD	Age, gender, residence, smoking, alcohol consumption, marital status, educational attainment, diabetes, CKD.
9	Ke Wang 2026 [23]	China	Average age: 58.99 ± 9.21 years	47.06%	6,715	TyG-WHtR	9 years	1.46 (1.31, 1.62)	Prospective cohort study	CVD	Age, gender, education level, CESD-10 score, sleep time, smoking, alcohol consumption, hypertension, diabetes, dyslipidemia and chronic kidney diseases.

*Abbreviations: BUN* Blood urea nitrogen, *Scr* Serum creatinine, *eGFR* Estimated glomerular filtration rate, *TC* Total cholesterol, *HDL-C* High-density lipoprotein cholesterol, *LDL-C* Low-density lipoprotein cholesterol, *CRP* C-reactive protein, *UA* Uric acid, *PLT* Platelet count, *HbA1c* Glycated hemoglobin, *CVD* Cardiovascular disease

### Quality assessment

The methodological quality of all included prospective cohort studies was evaluated using the Newcastle–Ottawa Scale (NOS), with a maximum total score of 9. Two studies achieved the maximum score of 9, while the remaining seven studies each scored 8. All included studies met the pre-specified quality threshold of ≥ 7 points and were classified as high-quality. The main reasons for score deductions among non-full-score studies included limitations in outcome ascertainment. Any discrepancies during quality assessment were resolved through consensus discussion. Detailed NOS scores for each individual study are presented in Table 2.

**Table 2:** Details of quality evaluation via the Newcastle–Ottawa Scale

Study (Publication Year)	Selection of cohorts			Comparability of cohorts			Outcome of cohorts			Total
	Representativeness of the exposed cohort	Selection of the non-exposed cohort	Ascertainment of exposure	Outcome not present at baseline	Control for age	Control for other confounding factors	Assessment of outcome	Sufficient follow-up duration	Adequacy of follow-up of cohorts	
Weipeng Li 2024 [15]	1	1	1	1	1	1	1	1	1	9
Kun Liu 2025 [16]	1	1	1	1	1	1	1	1	1	9
Jianan Hong 2025 [17]	1	1	1	1	1	1	0	1	1	8
Yilin Pan 2025 [18]	1	1	1	1	1	1	0	1	1	8
Menghe Wang 2025 [19]	1	1	1	1	1	1	0	1	1	8
Jintao Chen 2025 [20]	1	1	1	1	1	1	0	1	1	8
Cheng Huang 2025 [21]	1	1	1	1	1	1	0	1	1	8
Shu-Shu Han 2026 [22]	1	1	1	1	1	1	0	1	1	8
Ke Wang 2026 [23]	1	1	1	1	1	1	0	1	1	8

### Meta-analysis results

#### TyG index and risk of incident CVD

**Fig. 2:**
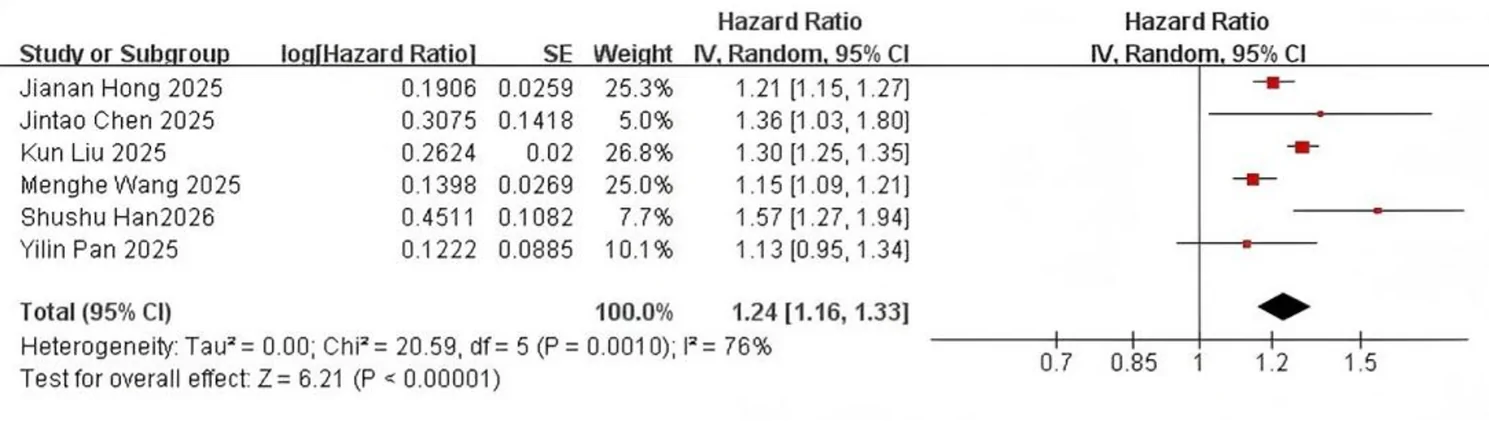
Forest plot of the association between TyG and CVD risk

A total of 6 observational cohort studies were incorporated into this meta-analysis focusing on the association between TyG index and incident CVD risk among patients with CKM syndrome stages 0–3. The overall pooled HR was 1.24 (95% CI: 1.16–1.33, I² = 76%; *P* < 0.001), which suggested a significant positive association between elevated TyG levels and higher incident CVD risk. Substantial between-study heterogeneity was observed across included studies (I² = 76%, χ² = 20.59, df = 5, *P* = 0.001). A random-effects model was adopted for effect size pooling. The forest plot is presented in Fig. 2.

To investigate potential sources of high heterogeneity across included studies, predefined subgroup analyses were performed stratified by participant age, follow-up duration, and total sample size. When stratified by age, the subgroup aged ≥ 60 years had a pooled HR of 1.18 (95% CI: 1.14–1.23, *P* < 0.00001) with trivial heterogeneity (I² = 3%). The subgroup aged < 60 years yielded a pooled HR of 1.39 (95% CI: 1.16–1.65, *P* = 0.0003) with moderate-to-high heterogeneity (I² = 66%). No statistically significant between-subgroup difference was detected (χ² = 3.07, df = 1, *P* = 0.08; I² = 67.4%). The corresponding forest plots are shown in Supplementary Material S3A. For subgroup analysis by follow-up duration, the subgroup with follow-up ≥ 8 years had a pooled HR of 1.29 (95% CI: 1.19–1.40, *P* < 0.00001) with substantial heterogeneity (I² = 77%). The subgroup with follow-up < 8 years presented a pooled HR of 1.15 (95% CI: 1.10–1.21, *P* < 0.00001) with negligible heterogeneity (I² = 0%). A significant difference was observed between the two subgroups (χ² = 5.08, df = 1, *P* = 0.02; I² = 80.3%). The relevant forest plots are displayed in Supplementary Material S3B. When stratified by total sample size, the large-sample subgroup (≥ 100,000 participants) had a pooled HR of 1.22 (95% CI: 1.09–1.38, *P* = 0.0010) with marked heterogeneity (I² = 93%). The small-sample subgroup (< 100,000 participants) showed a pooled HR of 1.27 (95% CI: 1.13–1.44, *P* < 0.0001) with moderate heterogeneity (I² = 56%). No significant between-subgroup difference was found (χ² = 0.19, df = 1, *P* = 0.66; I² = 0%). The corresponding forest plots are illustrated in Supplementary Material S3C.

#### TyG-BMI index and risk of incident CVD

**Fig. 3:**
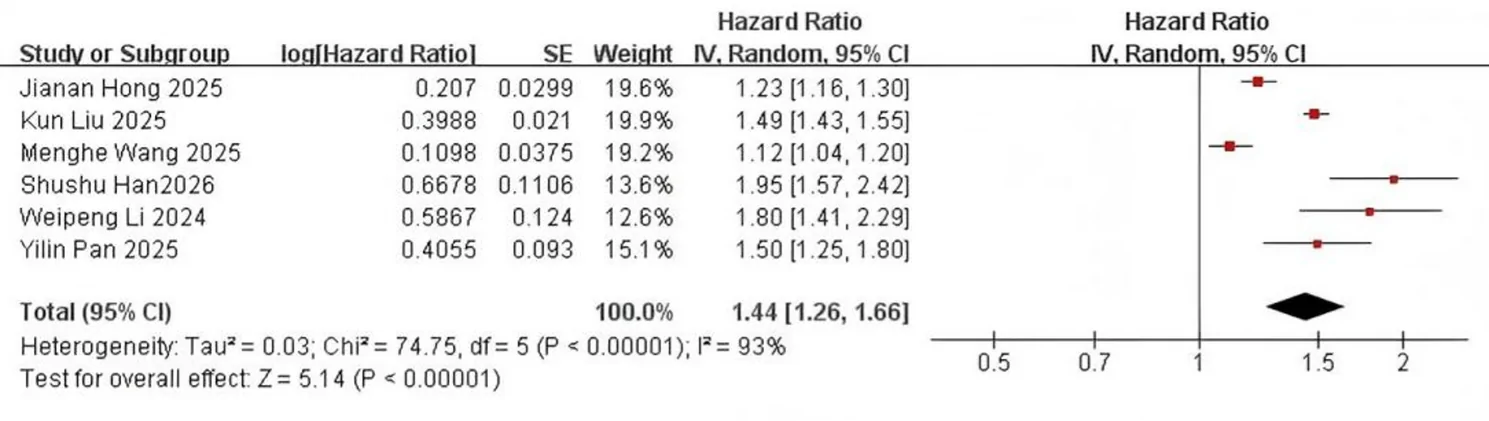
Forest plot of the association between TyG-BMI and CVD risk

A total of 6 observational cohort studies were incorporated into this meta-analysis focusing on the association between TyG-BMI and incident CVD risk among patients with CKM syndrome stages 0–3. The overall pooled HR was 1.44 (95% CI: 1.26–1.66, I² = 93%; *P* < 0.001), which suggested a significant positive association between elevated TyG-BMI levels and higher incident CVD risk. Marked between-study heterogeneity was observed across included studies (I² = 93%, χ² = 74.75, df = 5, *P* < 0.00001). A random-effects model was adopted for effect size pooling. The forest plot is presented in Fig. 3.

To investigate potential sources of high heterogeneity across included studies, predefined subgroup analyses were performed stratified by participant age, follow-up duration, and total sample size. When stratified by age, the subgroup aged ≥ 60 years had a pooled HR of 1.24 (95% CI: 1.10–1.39, *P* = 0.0004) with substantial heterogeneity (I² = 80%). The subgroup aged < 60 years yielded a pooled HR of 1.69 (95% CI: 1.40–2.05, *P* < 0.00001) with substantial heterogeneity (I² = 74%). A statistically significant between-subgroup difference was detected (χ² = 7.56, df = 1, *P* = 0.006; I² = 86.8%). The corresponding forest plots are shown in Supplementary Material 4 A. For subgroup analysis by follow-up duration, the subgroup with follow-up ≥ 8 years had a pooled HR of 1.54 (95% CI: 1.31–1.81, *P* < 0.00001) with marked heterogeneity (I² = 93%). The subgroup with follow-up < 8 years presented a pooled HR of 1.28 (95% CI: 0.96–1.71, *P* = 0.10) with marked heterogeneity (I² = 89%). No significant difference was observed between the two subgroups (χ² = 1.19, df = 1, *P* = 0.28; I² = 15.9%). The relevant forest plots are displayed in Supplementary Material 4B. When stratified by total sample size, the large-sample subgroup (≥ 100,000 participants) had a pooled HR of 1.29 (95% CI: 0.97–1.71, *P* = 0.08) with marked heterogeneity (I² = 98%). The small-sample subgroup (< 100,000 participants) showed a pooled HR of 1.57 (95% CI: 1.24–1.99, *P* = 0.0002) with marked heterogeneity (I² = 88%). No significant between-subgroup difference was found (χ² = 1.07, df = 1, *P* = 0.30; I² = 6.2%). The corresponding forest plots are illustrated in Supplementary Material 4 C.

#### TyG-WC index and risk of incident CVD

**Fig. 4:**
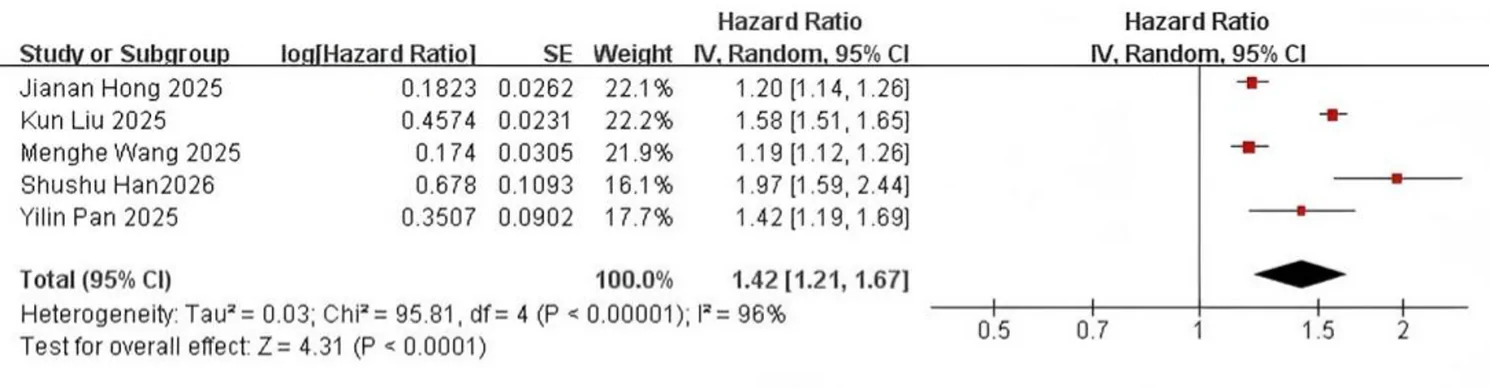
Forest plot of the association between TyG-WC and CVD risk

A total of 5 observational cohort studies were incorporated into this meta-analysis focusing on the association between TyG-WC and incident CVD risk among patients with CKM syndrome stages 0–3. The overall pooled HR was 1.42 (95% CI: 1.21–1.67, I² = 96%; *P* < 0.001), which suggested a significant positive association between elevated TyG-WC levels and higher incident CVD risk. Marked between-study heterogeneity was observed across included studies (I² = 96%, χ² = 95.81, df = 4, *P* < 0.00001). A random-effects model was adopted for effect size pooling. The forest plot is presented in Fig. 4.

To investigate potential sources of high heterogeneity across included studies, predefined subgroup analyses were performed stratified by participant age, follow-up duration, and total sample size. When stratified by age, the subgroup aged ≥ 60 years had a pooled HR of 1.30 (95% CI:1.12–1.51, *P* < 0.001) with I²=92%. The subgroup aged < 60 years yielded a pooled HR of 1.58 (95% CI:1.33–1.87, *P* < 0.001) with I²=93%. No statistically significant between-subgroup difference was detected (χ² =2.11, df = 1, *P* = 0.15; I²=52.6%). The corresponding forest plots are shown in Supplementary Material 5 A. For subgroup analysis by follow-up duration, the subgroup with follow-up ≥ 8 years had a pooled HR of 1.49 (95% CI:1.22–1.82, *P* < 0.001) with I²=94%. The subgroup with follow-up < 8 years presented a pooled HR of 1.32 (95% CI:1.05–1.66, *P* = 0.02) with I²=92%. No significant difference was observed between the two subgroups (χ² =1.03, df = 1, *P* = 0.31; I² = 2.9%). The relevant forest plots are displayed in Supplementary Material 5B. When stratified by total sample size, the large-sample subgroup (≥ 100,000 participants) had a pooled HR of 1.35 (95% CI:1.11–1.64, *P* = 0.003) with I²=95%. The small-sample subgroup (< 100,000 participants) showed a pooled HR of 1.48 (95% CI:1.21–1.82, *P* < 0.001) with I²=94%. No significant between-subgroup difference was found (χ² =0.69, df = 1, *P* = 0.41; I² = 0%). The corresponding forest plots are illustrated in Supplementary Material 5 C.

#### TyG-WHtR index and risk of incident CVD

**Fig. 5:**
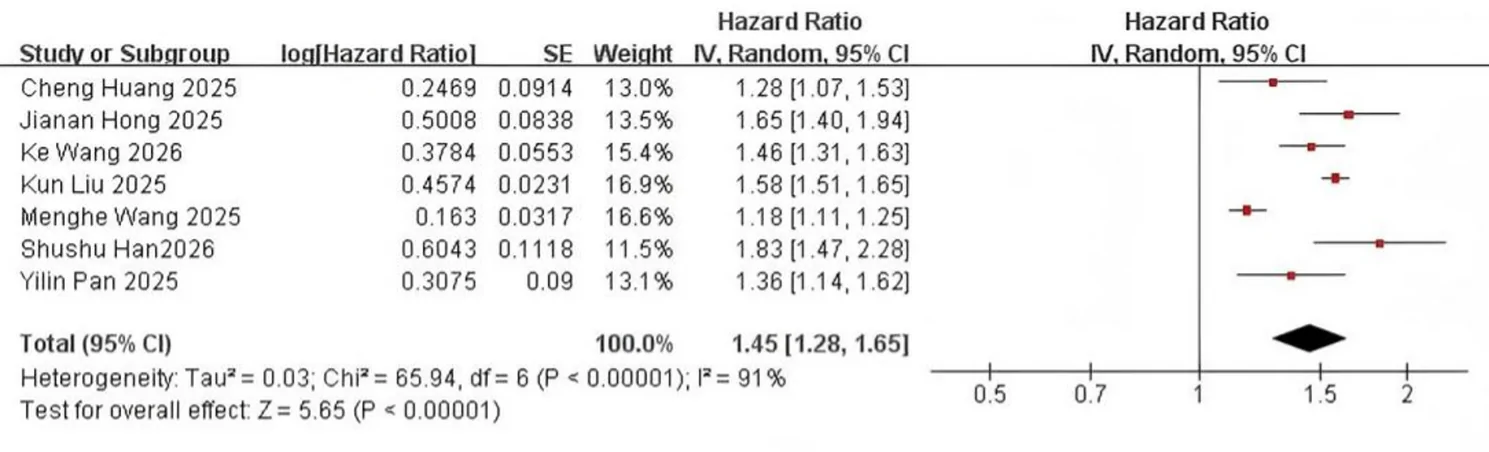
Forest plot of the association between TyG-WHtR and CVD risk

A total of 7 observational cohort studies were incorporated into this meta-analysis focusing on the association between TyG-WHtR and incident CVD risk among patients with CKM syndrome stages 0–3. The overall pooled HR was 1.45 (95% CI: 1.28–1.65, I² = 91%; *P* < 0.001), which suggested a significant positive association between elevated TyG-WHtR levels and higher incident CVD risk. Marked between-study heterogeneity was observed across included studies (I² = 91%, χ² = 65.94, df = 6, *P* < 0.00001). A random-effects model was adopted for effect size pooling. The forest plot is presented in Fig. 5.

To investigate potential sources of high heterogeneity across included studies, predefined subgroup analyses were performed stratified by participant age, follow-up duration, and total sample size. When stratified by age, the subgroup aged ≥ 60 years had a pooled HR of 1.32 (95% CI: 1.16–1.50, *P* < 0.001) with substantial heterogeneity (I² = 87%). The subgroup aged < 60 years yielded a pooled HR of 1.59 (95% CI: 1.32–1.92, *P* < 0.001) with marked heterogeneity (I² = 92%). No statistically significant between-subgroup difference was detected (χ² = 2.47, df = 1, *P* = 0.12; I² = 59.5%). The corresponding forest plots are shown in Supplementary Material 6 A. For subgroup analysis by follow-up duration, the subgroup with follow-up ≥ 8 years had a pooled HR of 1.51 (95% CI: 1.29–1.76, *P* < 0.001) with marked heterogeneity (I² = 93%). The subgroup with follow-up < 8 years presented a pooled HR of 1.34 (95% CI: 1.10–1.63, *P* = 0.004) with substantial heterogeneity (I² = 85%). No significant difference was observed between the two subgroups (χ² = 1.36, df = 1, *P* = 0.24; I² = 26.3%). The relevant forest plots are displayed in Supplementary Material 6B. When stratified by total sample size, the large-sample subgroup (≥ 100,000 participants) had a pooled HR of 1.36 (95% CI: 1.14–1.62, *P* < 0.001) with marked heterogeneity (I² = 92%). The small-sample subgroup (< 100,000 participants) showed a pooled HR of 1.51 (95% CI: 1.27–1.79, *P* < 0.001) with marked heterogeneity (I² = 91%). No significant between-subgroup difference was found (χ² = 1.15, df = 1, *P* = 0.28; I² = 12.9%). The corresponding forest plots are illustrated in Supplementary Material 6 C.

### Publication bias analysis

Publication bias was evaluated for all four TyG-derived indices (TyG, TyG-BMI, TyG-WC, and TyG-WHtR) via visual inspection of funnel plots and formal Egger’s linear regression tests.

**Fig. 6:**
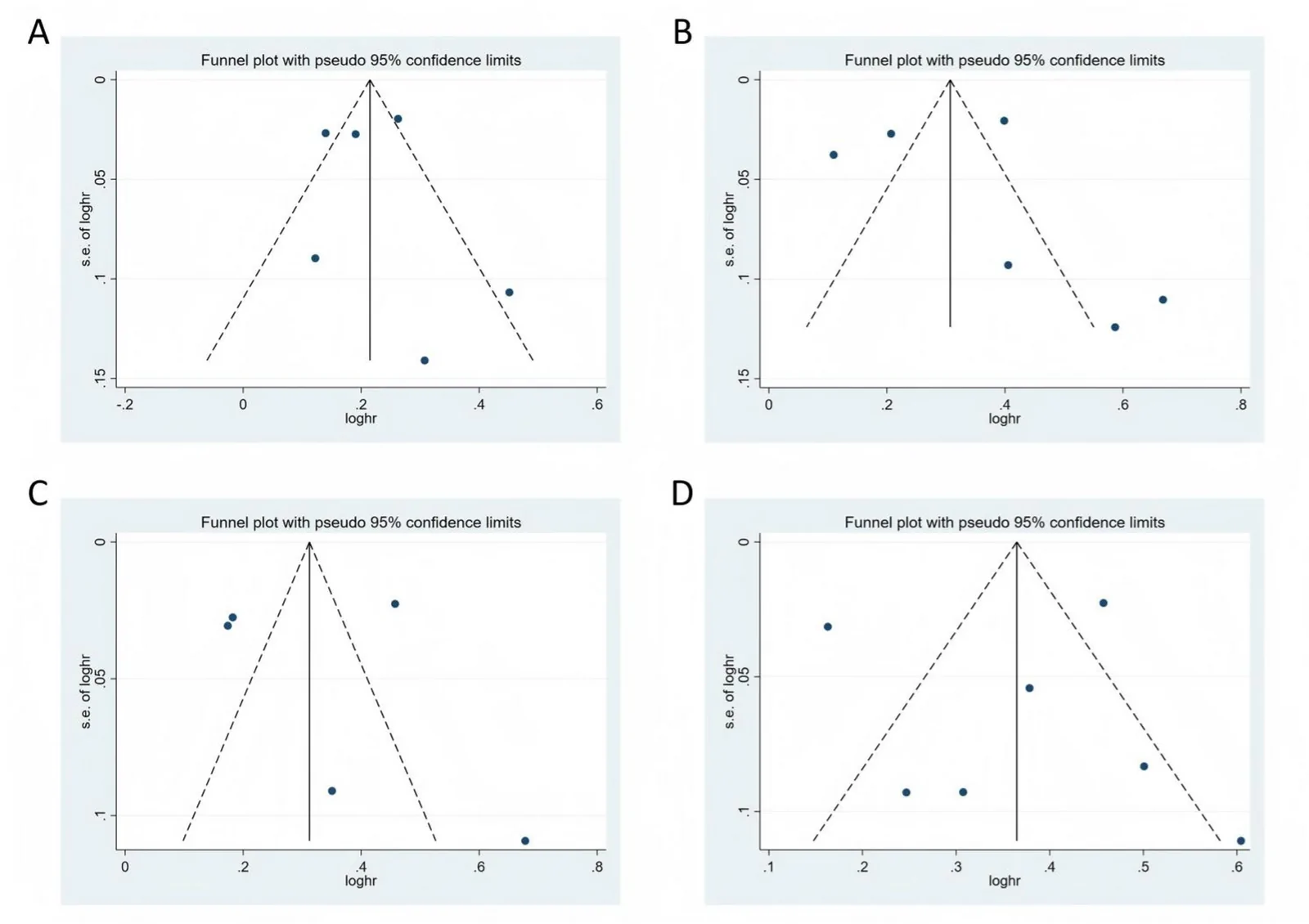
Funnel plots testing publication bias for four TyG indices and CVD risk: (**A**) TyG; (**B**) TyG-BMI; (**C**) TyG-WC; (**D**) TyG-WHtR

Egger’s regression analysis detected no statistically significant intercept for any of the four indices (all *P* > 0.05), with specific results as follows: TyG (intercept = 0.3049, *P* = 0.865), TyG-BMI (intercept = 1.1401, *P* = 0.741), TyG-WC (intercept = 0.8680, *P* = 0.882), and TyG-WHtR (intercept = − 0.0861, *P* = 0.975). These findings provided no statistical evidence of significant publication bias. Visual assessment of funnel plots further supported this result, as individual study effect estimates were approximately symmetrically distributed around the pooled log-transformed HR for each index. The funnel plots for TyG, TyG-BMI, TyG-WC, and TyG-WHtR are presented in Fig. 6A, B and C, and 6D, respectively.

Notably, as fewer than 10 studies were included for each TyG-derived index, the statistical power of publication bias detection was inherently limited. Therefore, these results should be interpreted with caution, and the presence of occult publication bias cannot be definitively ruled out.

### Sensitivity analysis

Leave-one-out sensitivity analyses were performed to evaluate the robustness of the pooled associations between the four TyG-derived indices and incident CVD risk. For the TyG index: sequential omission of each individual study yielded pooled HRs ranging from 1.22 to 1.26, with all recalculated 95% CIs remaining entirely above the null value of 1 and the combined effects consistently statistically significant (all *P* < 0.001). For TyG-BMI: after excluding each study one at a time, the pooled HR fluctuated between 1.37 and 1.51, with all 95% CIs not crossing the null value and statistical significance preserved (all *P* < 0.001). For TyG-WC: the recalculated pooled HRs varied from 1.33 to 1.50, with all effect estimates remaining statistically significant and corresponding 95% CIs entirely above 1. For TyG-WHtR: sequential omission of each eligible study resulted in pooled HRs ranging from 1.41 to 1.52, with all pooled estimates retaining statistical significance and 95% CIs consistently greater than 1. Overall, no material changes in pooled effect sizes or statistical significance were observed for any of the four indices after single-study exclusion, confirming that the primary meta-analysis results were statistically robust. Detailed sensitivity analysis results are presented in Table 3.

**Table 3:** Summary of sensitivity analysis

Dataset excluded	HR	95%CI	I^2^%	*P* for effect
TyG and incident CVD risk				
Jianan Hong 2025 [17]	1.26	[1.14, 1.39]	80	*P* < 0.001
Jintao Chen 2025 [20]	1.24	[1.15, 1.33]	80	*P* < 0.001
Kun Liu 2025 [16]	1.22	[1.13, 1.31]	59	*P* < 0.001
Menghe Wang 2025 [19]	1.27	[1.18, 1.36]	63	*P* < 0.001
Shushu Han 2026 [22]	1.22	[1.14, 1.30]	75	*P* < 0.001
Yilin Pan 2025 [18]	1.26	[1.17, 1.35]	79	*P* < 0.001
TyG-BMI and incident CVD risk				
Jianan Hong 2025 [17]	1.51	[1.26, 1.81]	93	*P* < 0.001
Kun Liu 2025 [16]	1.44	[1.22, 1.69]	89	*P* < 0.001
Menghe Wang 2025 [19]	1.53	[1.33, 1.76]	90	*P* < 0.001
Shushu Han 2026 [22]	1.37	[1.19, 1.59]	94	*P* < 0.001
Weipeng Li 2024 [15]	1.40	[1.21, 1.62]	94	*P* < 0.001
Yilin Pan 2025 [18]	1.44	[1.23, 1.68]	95	*P* < 0.001
TyG-WC and incident CVD risk				
Jianan Hong 2025 [17]	1.49	[1.22, 1.82]	95	*P* < 0.001
Kun Liu 2025 [16]	1.35	[1.19, 1.54]	87	*P* < 0.001
Menghe Wang 2025 [19]	1.50	[1.23, 1.82]	96	*P* < 0.001
Shushu Han 2026 [22]	1.33	[1.13, 1.58]	96	*P* = 0.0007
Yilin Pan 2025 [18]	1.42	[1.19, 1.70]	97	*P* = 0.0001
TyG-WHtR and incident CVD risk				
Cheng Huang 2025 [21]	1.48	[1.28, 1.70]	92	*P* < 0.001
Jianan Hong 2025 [17]	1.42	[1.23, 1.64]	92	*P* < 0.001
Ke Wang 2026 [23]	1.45	[1.25, 1.69]	92	*P* < 0.001
Kun Liu 2025 [16]	1.43	[1.24, 1.64]	84	*P* < 0.001
Menghe Wang 2025 [19]	1.52	[1.40, 1.64]	55	*P* < 0.001
Shushu Han 2026 [22]	1.41	[1.23, 1.61]	92	*P* < 0.001
Yilin Pan 2025 [18]	1.47	[1.27, 1.69]	92	*P* < 0.001

### Meta-regression analyses

Exploratory univariable meta-regression analyses were conducted to investigate potential moderators associated with between-study heterogeneity. Follow-up duration was significantly associated with heterogeneity in the pooled effect estimates of the TyG index (β = 0.0139, *P* = 0.047), whereas participant age was significantly associated with heterogeneity in the pooled effect estimates of the TyG-WHtR index (β=-0.0203, *P* = 0.036). No significant moderators were identified for the TyG-BMI or TyG-WC indices. Detailed results are presented in Table 4.

**Table 4:** Exploratory univariable meta-regression analyses of potential moderators for TyG-derived indices

Index	Moderator	β (coefficient)	95% CI	*P* value
TyG	Age	-0.0087	-0.0207, 0.0033	0.114
TyG	Follow-up duration	0.0139	0.0003, 0.0275	0.047
TyG	ln(Sample size)	-0.0137	-0.0758, 0.0484	0.573
TyG-BMI	Age	-0.0279	-0.0673, 0.0115	0.121
TyG-BMI	Follow-up duration	0.0157	-0.0758, 0.1071	0.659
TyG-BMI	ln(Sample size)	-0.0533	-0.1679, 0.0613	0.266
TyG-WC	Age	-0.0226	-0.0739, 0.0287	0.255
TyG-WC	Follow-up duration	0.0216	-0.0802, 0.1234	0.548
TyG-WC	ln(Sample size)	-0.0271	-0.1818, 0.1277	0.616
TyG-WHtR	Age	-0.0203	-0.0387, -0.0019	0.036
TyG-WHtR	Follow-up duration	0.0302	-0.0126, 0.0730	0.130
TyG-WHtR	ln(Sample size)	-0.0276	-0.1043, 0.0491	0.398

*β* Regression coefficient, *CI* Confidence interval. Meta-regression analyses were conducted using the restricted maximum likelihood (REML) approach

## Discussion

This systematic review and meta-analysis synthesized evidence from nine high-quality prospective cohort studies to systematically evaluate the associations of four TyG-derived indices (TyG, TyG-BMI, TyG-WC, and TyG-WHtR) with incident CVD risk in individuals with stage 0–3 CKM syndrome. The main findings indicated significant positive associations between elevated levels of all four TyG-derived indices and increased CVD risk in this population. Compared with the single TyG index (HR = 1.24), the three anthropometric composite indices showed numerically larger pooled effect estimates, with pooled HRs of 1.44 for TyG-BMI, 1.42 for TyG-WC, and 1.45 for TyG-WHtR. However, these numerical differences should not be interpreted as evidence of superiority because direct statistical comparisons between indices were not performed and confidence intervals overlapped. Leave-one-out sensitivity analyses supported the stability of the pooled estimates, and no significant publication bias was detected; however, these findings should be interpreted cautiously given the limited number of studies available for each index.

Pre-specified subgroup analyses were conducted to explore potential sources of between-study heterogeneity. For the TyG index, follow-up duration was identified as a potential moderator of heterogeneity: cohorts with longer follow-up (≥ 8 years) showed slightly stronger risk estimates, which may reflect the possibility that longer follow-up periods capture the cumulative effects of persistent metabolic abnormalities on cardiovascular outcomes. For TyG-BMI, age-stratified analysis suggested a more pronounced association in individuals aged < 60 years, which may indicate that obesity-related metabolic disturbances could have different implications across age groups [24]. However, these subgroup findings should be interpreted as exploratory and require further validation in larger dedicated cohorts. In contrast, no statistically significant subgroup differences were observed for TyG-WC and TyG-WHtR across age, follow-up duration, and sample size strata.

The observed associations may be partially explained by pathophysiological pathways involving insulin resistance [2, 25]. As a well-validated surrogate marker of systemic insulin resistance, the TyG index reflects abnormalities in glucose and triglyceride metabolism. Individuals with CKM syndrome may exhibit metabolic and renal abnormalities that contribute to impaired glycolipid homeostasis and further aggravate insulin resistance [26]. Increased insulin resistance may subsequently impair vascular endothelial function, reduce nitric oxide-mediated vasodilation, and promote persistent vascular inflammation and oxidative stress. These pathological changes may facilitate atherosclerotic processes and contribute to an increased risk of cardiovascular events [27–29].

Beyond isolated glycolipid disturbance, composite TyG indices integrate obesity-related anthropometric parameters, potentially providing a more integrated assessment of cardiometabolic burden. TyG-BMI, TyG-WC, and TyG-WHtR respectively reflect the combined burden of insulin resistance with general obesity, abdominal obesity, and body-size-adjusted central adiposity [30, 31]. Visceral fat accumulation in individuals with CKM syndrome may further contribute to lipotoxicity, chronic low-grade inflammation, and metabolic dysregulation, thereby promoting adverse cardiovascular alterations. These mechanisms may partially explain the numerically higher pooled estimates observed for composite indices compared with the single TyG index [32, 33]. However, these numerical differences should not be interpreted as evidence of superiority because direct statistical comparisons between indices were not performed [32, 33].

Cardiorenal metabolic crosstalk may provide a potential framework for understanding why TyG-related metabolic abnormalities are particularly relevant to CVD risk in individuals with CKM syndrome [1]. Although early-stage CKM syndrome may not involve overt renal failure, subtle metabolic and renal abnormalities may already be present in some individuals, potentially affecting lipid and glucose homeostasis [34]. These metabolic disturbances may reinforce a vicious cycle involving impaired glycolipid metabolism and insulin resistance, thereby further increasing cardiometabolic burden [35]. Additionally, CKM-related metabolic disturbances may be accompanied by chronic inflammation and oxidative stress, which may contribute to renal and cardiovascular pathological changes. In this context, TyG-derived indices may represent measurable markers reflecting the relationship between early metabolic abnormalities and subsequent cardiovascular risk [36].

From a methodological perspective, several notable strengths underpin the credibility of the pooled findings in this meta-analysis. First, all included prospective cohort studies achieved high NOS scores (8–9 points), reflecting generally robust study design, clearly defined outcome assessment, and adjustment for relevant confounding factors. These methodological characteristics may reduce the risk of major bias and enhance the credibility of the synthesized evidence. Second, leave-one-out sensitivity analyses revealed no material changes in pooled effect estimates or statistical significance after sequential exclusion of individual studies, suggesting that the overall associations were not materially influenced by any single cohort and supporting the robustness of the pooled estimates.

In clinical practice, TyG-derived indices may serve as low-cost and readily available auxiliary markers for preliminary CVD risk stratification. However, whether any specific composite index provides superior risk stratification performance requires direct comparative studies. These markers may help identify individuals with higher cardiometabolic risk who may not be fully captured by conventional CVD risk assessment approaches, thereby facilitating earlier risk assessment and preventive strategies. Given that the current evidence is derived from observational cohort studies, whether TyG-derived indices play a causal role in CVD development remains uncertain. Further large-scale, multi-ethnic prospective cohort studies are warranted to validate these findings and establish clinically applicable risk stratification thresholds for CKM populations.

## Strengths and limitations

### Strengths

To the best of our knowledge, this is the first systematic review and meta-analysis to systematically evaluate the associations of four core TyG-derived indices (TyG, TyG-BMI, TyG-WC, and TyG-WHtR) with incident CVD risk specifically in individuals with stage 0–3 CKM syndrome, providing population-specific quantitative evidence for cardiovascular risk stratification and addressing an important evidence gap in this field. Second, all included studies were high-quality prospective cohort studies with NOS scores of 8–9, supporting the methodological quality of the included evidence and enhancing confidence in the synthesized findings. Third, rigorous statistical analyses were applied throughout the study. Pre-specified subgroup analyses stratified by age, follow-up duration, and sample size were conducted to explore potential sources of heterogeneity; leave-one-out sensitivity analyses supported the stability of pooled effect estimates; and funnel plot visualization combined with Egger’s regression test was used to evaluate potential publication bias. Fourth, all TyG-derived indices included in this analysis are simple, low-cost, and easily obtainable in routine clinical practice, suggesting potential utility as auxiliary markers for preliminary cardiovascular risk stratification.

### Limitations

Several limitations of this meta-analysis should be acknowledged. First, substantial between-study heterogeneity was observed across all four index analyses, which may reflect variations in baseline population characteristics, follow-up duration, CKM and CVD event definitions, and adjusted confounding variables across individual studies. Although all included studies adopted the CKM syndrome framework and enrolled individuals classified within stage 0–3 CKM syndrome according to cohort-specific criteria, differences in the operational definitions and assessment procedures of CKM components across cohorts could not be completely excluded and may have contributed to residual heterogeneity. Pre-specified subgroup analyses and exploratory univariable meta-regression analyses were performed to investigate potential moderators of heterogeneity; however, residual unexplained heterogeneity remained. Furthermore, the limited number of studies available for each TyG-derived index prevented the implementation of multivariable meta-regression analyses because of the potential risk of model overfitting and unstable estimates.

Second, the number of included studies per index was limited (ranging from 5 to 7, all fewer than 10). In accordance with meta-analytic guidelines, the statistical power of Egger’s test for detecting publication bias is limited when the number of included studies is small; therefore, potential undetected or unreported publication bias cannot be completely ruled out.

Third, all enrolled studies adopted an observational cohort design. This study observed significant associations between elevated TyG-derived indices and increased CVD risk among individuals with CKM syndrome, but cannot establish a definitive causal relationship. Therefore, the findings should be interpreted cautiously, and causal inferences require confirmation from further prospective investigations and mechanistic studies.

Fourth, confounding adjustment strategies were inconsistent across included studies, and some unmeasured residual confounders, such as lifestyle factors, dietary patterns, and other metabolic-related factors, may still have influenced the pooled effect estimates.

Finally, the included studies were predominantly conducted in Chinese populations, with only one study from the United Kingdom, which may limit the generalizability of the findings. Further validation in large-scale multi-ethnic and multi-regional cohorts is warranted.

## Conclusions

In conclusion, this meta-analysis suggests that elevated levels of all four TyG-derived indices are significantly associated with increased incident CVD risk among individuals with stage 0–3 CKM syndrome. Anthropometric composite indices showed numerically higher pooled effect estimates than the single TyG index; however, the overlapping confidence intervals and lack of direct statistical comparisons preclude determining whether any specific index is superior to others. The pooled estimates remained stable in leave-one-out sensitivity analyses, and no significant publication bias was detected, although the statistical power of these tests was limited by the small number of studies available for each index. Exploratory subgroup and meta-regression analyses suggested that certain study-level characteristics, such as age and follow-up duration, may partially contribute to variations in effect estimates across different TyG-derived indices. Given their low cost and accessibility, TyG-derived indices may serve as potential auxiliary markers for CVD risk stratification in individuals with early-stage CKM syndrome. Further large-scale multi-ethnic prospective cohort studies and mechanistic investigations are warranted to validate these findings and clarify their clinical utility.

## Supplementary Information

Supplementary Material 1.

## Data Availability

All the original data used in this study is included in the article and supplementary materials.

## Abbreviations

TyG: Triglyceride-glucose index
TyG-BMI: TyG-body mass index
TyG-WC: TyG-waist circumference
TyG-WHtR: TyG-waist-to-height ratio
CKM: Cardio-kidney-metabolic
CVD: Cardiovascular disease
HR: Hazard ratio
CI: Confidence interval
NOS: Newcastle-Ottawa Scale
